# Rice Sesquiterpene Plays Important Roles in Antixenosis against Brown Planthopper in Rice

**DOI:** 10.3390/plants10061049

**Published:** 2021-05-22

**Authors:** Wintai Kamolsukyeunyong, Wissarut Sukhaket, Kitsada Pitija, Pornwalai Thorngkham, Sugunya Mahatheeranont, Theerayut Toojinda, Apichart Vanavichit

**Affiliations:** 1 National Center for Genetic Engineering and Biotechnology (BIOTEC), 113 Thailand Science Park, Phahonyothin Road, Khlong Nueng, Khlong Luang, PathumThani 12120, Thailand; wtkmsyy@gmail.com (W.K.); theerayut@biotec.or.th (T.T.); 2 Expert Center of Innovative Agriculture, Thailand Institute of Scientific and Technological Research (TISTR), 35 Mu 3 Technopolis, Khlong Ha, Khlong Luang, Pathum Thani 12120, Thailand; wissarut_suk@tistr.or.th; 3 Department of Chemistry, Faculty of Science, Chiang Mai University, Chiang Mai 50200, Thailand; kitsadapiti@gmail.com (K.P.); finsofia@yahoo.com (P.T.); sugunya.m@cmu.ac.th (S.M.); 4 Research Center on Chemistry for the Development of Health-Promoting Products from Northern Resources, Chiang Mai University, Chiang Mai 50200, Thailand; 5 Department of Agronomy, Faculty of Agriculture at Kamphaeng Saen, Kamphaeng Saen Campus, Kasetsart University, Nakhon Pathom 73140, Thailand; 6 Rice Science Center, Kasetsart University, Kamphaeng Saen, Nakhon Pathom 73140, Thailand

**Keywords:** brown planthopper, terpene synthase, E-β-farnesene, transcription factor binding site

## Abstract

The rice sesquiterpene synthase II gene (*OsSTPS2*, LOC_Os04g27430), which is involved in the antixenosis defense mechanism of rice against brown planthopper (BPH) infestation, was identified in the BPH-resistant rice variety Rathu Heenati (RH). In contrast, the gene was not functional in the BPH-susceptible rice variety KDML105 (KD). Single-nucleotide polymorphisms (SNPs) in the promoter region and in exon 5 of the gene and a seven amino acid deletion in the deduced protein sequence are suggested as factors that negatively regulate the function of the gene. Sequence analysis of the promoter region and expression analysis of the *OsSTPS2* gene in several rice genotypes revealed the correlation of SNPs of the ATHB-1, SBE1, and P-factor with the expression of the gene. Genomic and complementary DNA (cDNA) sequence analysis at exon 5 of the gene showed that the 21 bp deletion naturally occurred in several rice genotypes. The antixenosis of the BPH feeding preference (AFP) of rice varieties differed in the seven amino acid deletion lesion of the gene, suggesting that the seven amino acid deletion negatively controls the antixenosis mechanism during BPH infestation. Analysis of the plant volatile compounds released after BPH infestation suggested that E-β-farnesene (EBF) is the major product of the *OsSTPS2* gene.

## 1. Introduction

Due to the long-term evolutionary interactions between plants and their insect enemies, many plants release elevated levels of volatile organic compounds upon being attacked by herbivores. Some of these plant volatiles have functional roles in the plants’ defense mechanisms [1,2]. Terpenoids, including monoterpenes, sesquiterpenes, and diterpenes, are the most common and diverse group of insect-induced plant volatiles. All terpenoids are synthesized by the action of terpene synthases (TPSs), which convert geranyl diphosphate (GPP), farnesyl diphosphate (FPP), and geranylgeragyl diphosphate (GGPP) to monoterpenes, sesquiterpenes, and diterpenes, respectively [3,4]. Rice contains at least 45 terpene synthase (TPS) genes located in almost every chromosome of the genome (http://rice.plantbiology.msu.edu/cgi-bin/putative_function_search.pl; accessed on 21 May 2021). We previously identified LOC_Os04g27430 as the sesquiterpene synthase II (*OsSTPS2*) gene induced by brown planthopper (BPH) feeding in the BPH-resistant rice variety Rathu Heenati (RH) [5]. The gene was found to play a role in the antixenosis mechanism against BPH infestation. In addition, its haplotype patterns (HPs), along with another three BPH resistance genes (*OsLecRK2-3* and *BPH32*), have been found to play a role in broad-spectrum BPH resistance in RH-derived introgression lines (ILs) [6]. However, the major sesquiterpene volatile compound products of this gene have still not been identified. *OsSTPS2* was found to be reduced at both the genomic and protein levels in the rice variety KDML105 (KD), which is highly susceptible to BPH attack. Damage to this gene results from three single-nucleotide polymorphisms (SNPs) at transcription factor binding sites in the promoter region and a 21 bp deletion in the cDNA leading to a seven amino acid deletion in the protein chain.

In this study, we investigated the correlation between sequence variation in the promoter region of the *OsSTPS2* gene and its expression in several rice varieties/lines and showed that the gene may be controlled at the transcription level. We also found that the seven amino acid deletion in the polypeptide chain of the gene is a natural occurrence in tested rice panels. Finally, we identified E-β-farnesene as the candidate major sesquiterpene volatile compound product of this gene.

## 2. Results and Discussion

### 2.1. Expression of OsSTPS2 Is Associated with SNPs at Transcription Factor (TF) Binding Sites

**Figure 1:**
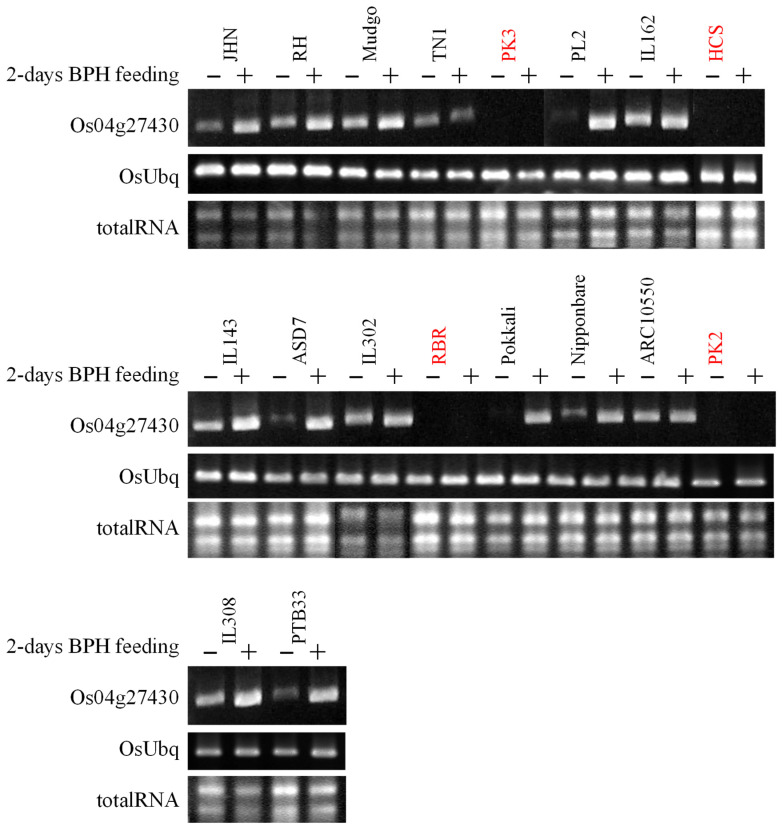
Expression pattern of *OsSTPS2* (Os04g27430) in control (−) and 2-day brown planthopper (BPH) feeding (+) conditions; rice varieties with an undetectable expression of the gene are labeled in red. The rice ubiquitin (*OsUbq*) was used as the housekeeping gene check.

The association between single-nucleotide polymorphisms (SNPs) in three transcription factor binding elements (TFBEs) and the expression of the *OsSTPS2* gene was investigated in the 18 rice varieties/lines. The TFBEs were included the *Arabidopsis thaliana* homeobox protein 1 (ATHB-1; consensus sequence AATTAATTATTGCT), silencer-binding factor 1 (SBE1; consensus sequence GTGTGGTTAATAAT), and maize activator P (P element; consensus sequence ACCAACCAG). The promoter region and 5’UTR of the *OsSTP2* gene were analyzed in 18 rice varieties/lines (NCBI accession no. KC511030 to KC511047). Four rice varieties, including Pinkaset2 (PK2), Pinkaset3 (PK3), Homchonlasit (HCS), and Riceberry (RBR), have all SNPs in the TFBEs, which silence the *OsSTP2* in either BPH feeding or control conditions (Figure 1, Table 1, Supplementary Figure S1). In contrast, the other 13 rice varieties/lines which express the *OsSTP2* gene inducibly do not have SNPs in the TFBEs. Correlation between SNPs in the TFBEs and the *OsSTPS2* expression suggests the roles of these TFBEs in the BPH inducible expression. Based on SNPs in these three TFBEs, three haplotype patterns (HP) of the *OsSTPS2* gene were identified in these 18 rice varieties/lines (Table 1). The rice genotypes with the HP1 of the *OsSTPS2* gene consisted of KD, PK2, PK3, RBR, and HCS. All of them were susceptible to BPH. The rice varieties that have *OsSTP2* expression were grouped into the HP2, consisted of Jaohomnin (JHN), Nipponbare, TN1, Mudgo, ASD7, ARC10550, and Pokkali. Finally, six rice varieties/lines, including PTB33, RH, IL143, IL162, IL302, and IL308, were grouped into the HP3. The *OsSTPS2* gene was also expressed in this HP group.

### 2.2. The Seven Amino Acid Deletion in OsSTPS2 Is a Natural Variation

The KD allele of the *OsSTPS2* gene contained two SNPs—TG > CA—in exon 5, leading to 21 bp and seven amino acid deletions in the mRNA and protein sequence, respectively. To investigate whether these two SNPs were found in other rice varieties, genomic DNA harboring this region of the gene was amplified from several rice varieties, and the sequences (accession no KP117047 to KP117059) were aligned. Interestingly, it was found that not only KD but also PK2, PK3, RBR, and HCS contained these two SNPs in exon 5 of the gene (Table 1, Figure S2). However, Mudgo, TN1, Pokkali, ARC10550, ASD7, JHN, and Nipponbare contained the TG nucleotides in the same manner as PTB33 and ILs, but the 21 bp deletion occurred in the genomic DNA of these varieties. The *OsSTPS2* genes expressed by nine rice varieties and three ILs (IL143, IL302, and IL308) were sequenced (accession no. KC511057 to KC511068). Interestingly, it was found that not only KD but also JHN, TN1, Mudgo, Pokkali, ARC10550, ASD7, and Nipponbare have this 21 bp deletion in their allele of the gene (Table 1, Figure S2). On the other hand, the 21 bp deletion was not found in the alleles of BPH-resistant varieties such as PTB33, Pitsanulok 2 (PL2), and all ILs tested. The deduced amino acid sequences of these varieties were translated, and the seven amino acid deletion was found in JHN, TN1, Mudgo, Pokkali, ARC10550, ASD7, and Nipponbare, respectively (Figure S2). This finding suggests that not only KD but some other rice varieties, both BPH-resistant and susceptible, also contain seven amino acid deleted lesions in the *OsSTPS2* gene.

### 2.3. Antixenosis Mechanism Is Not Functional in Rice Varieties Harboring a Seven Amino Acid Deletion in the OsSTPS2 Gene

**Figure 2:**
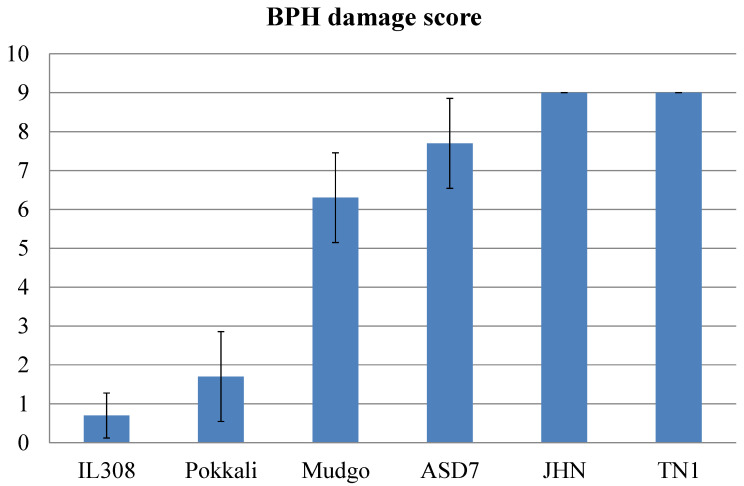
BPH damage score reaction against brown planthopper (BPH) population from Ubon Rachathani (UBN-BPH) for six rice varieties/lines at seven days after BPH infestation (7DAI). The error bars are the standard deviation (SD).

**Figure 3:**
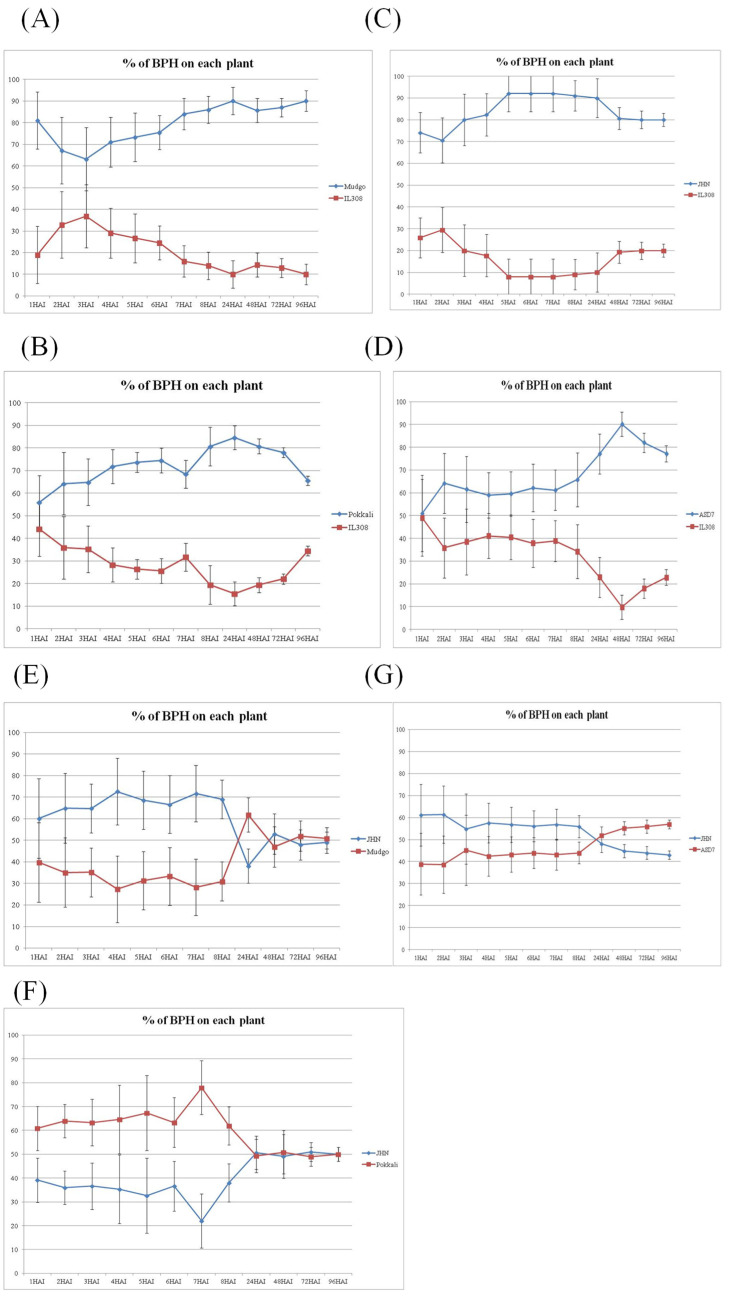
Antixenosis of BPH feeding preference (AFP) test. (**A**–**D**) Comparisons between IL308 and other varieties; (**E**–**G**) comparisons between JHN and other varieties. A total of 10 second- and third-instar BPH nymphs were allowed to settle on the experimental plants, and five replicates were performed for each comparison. The y axis indicates the number of BPHs that settled on each plant as a percentage, and the *x* axis shows the progress between 1 and 96 h after BPH infestation. The error bars are the standard error (SE). HAI = hours after BPH infestation.

To elucidate the effect of the seven amino acid deletion in the deduced protein sequence of the *OsSTPS2* gene on its putative function in the antixenosis of BPH feeding preference (AFP), five rice varieties/lines were selected for AFP study: JHN, Mudgo, ASD7, Pokkali, and IL308. The *OsSTPS2* gene was expressed in all these rice varieties/lines, but the seven amino acid deletion was found only in JHN, Mudgo, ASD7, and Pokkali. The BPH damage score reaction against the BPH population from Ubon Rachathani (UBN-BPH) showed that IL308 and Pokkali were BPH resistant, Mudgo and ASD7 were moderately susceptible, and JHN was BPH susceptible (Figure 2, Supplementary Table S1). The AFP comparison between IL308 and other rice varieties revealed that in all combinations, BPH preferred to settle on Mudgo, Pokkali, JHN, and ASD7 than on IL308, respectively (Figure 3A–D, Table S2). Even though both IL308 and Pokkali were BPH resistant (with scores of 0.7 and 1.7, respectively), the AFP test clearly demonstrated that, over the 96 h period of BPH infestation, BPHs preferred Pokkali to IL308. The AFP comparisons between JHN, the BPH-susceptible variety, and other rice varieties are shown in Figure 3E–G. Interestingly, the AFP comparison revealed that even though JHN was the most susceptible rice variety in this group, BPHs randomly settled on the tested plants over the 96 h period of the BPH settling choice test. The results from the AFP experiments demonstrated that the putative function of the *OsSTPS2* gene in the antixenosis mechanism is regulated not only at the transcription level by sequence variation at the promoter region of the gene, but also at the translation level in a process by which rice varieties containing the allele with the seven amino acid deletion were unable to proceed with the antixenosis mechanism during BPH infestation.

### 2.4. OsSTPS2 Is Responsible for the Production of E-β-Farnesene in RH and IL Infested by BPH

**Figure 4:**
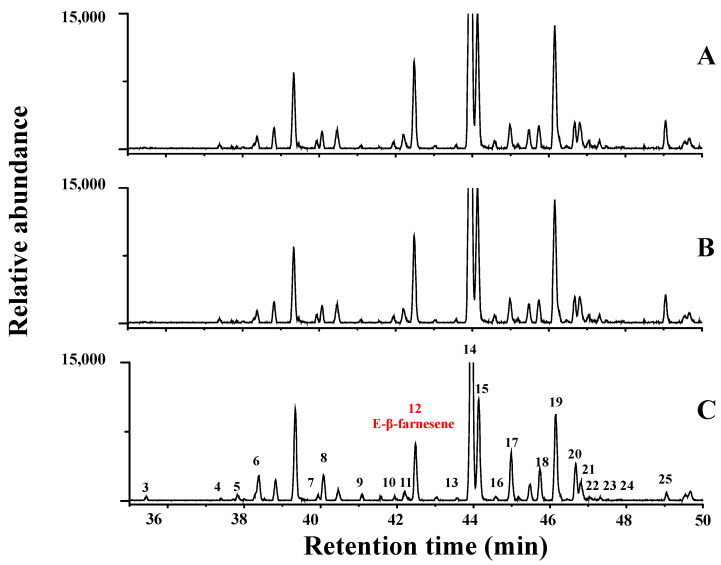
Sesquiterpene products of rice plants after 10 days of BPH feeding treatment. (**A**) = Rathu heenati, (**B**) = IL283, and (**C**) = KDML105. Peaks are as follows: 1, linalool; 2, isopulegol; 3, a-cubebene; 4, a-copaene; 5, b-cubebene; 6, b-elemene; 7, trans-caryophyllene; 8, epi-bicyclosesquiphellandrene; 9, a-bergamotene; 10, E-geranyl acetone; 11, a-humulene; 12, trans-b-farnesene; 13, g-muurolene; 14, b-ionone; 15, b-ionone epoxide; 16, germacrene B; 17, a-zingiberene; 18, b-bisabolene; 19, d-cadinene; 20, (-)-calamenene; 21, b-sesquiphellandrene; 22, trans-g-bisabolene; 23, 1,2,3,4,4a,7,-hexahydro-1,6-dimethyl-4-(1-methylethyl),naphthalene; 24, calacorene; and 25, nerolidol. Peak number 12 (tran or E-β-farnesene) is labeled in red.

**Figure 5:**
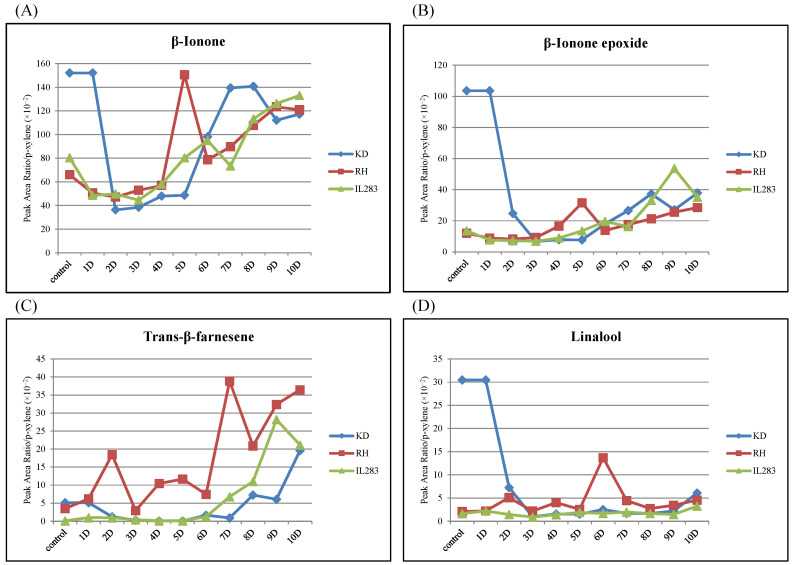
BPH-feeding-induced emission of the major sesquiterpenes. Changes of β-ionone (**A**), β-ionone epoxide (**B**), trans or E-β-farnesene (**C**), and linalool (**D**) emission after BPH feeding for 0–10 days in RH (red line), IL283 (green line), and KD (blue line). D = Days after brown planthopper (BPH) infestation.

Sesquiterpenes are the major plant volatile compounds released after damage by herbivore infestation [7,8,9]. The production of multiple volatile compound products in response to the expression of one sesquiterpene synthase gene is a natural phenomenon found in several plant species [10,11,12]. A total of 25 sesquiterpenes were identified by GC-MS in the mixture of volatile compounds emitted by KD, IL283, and RH rice plants infested by BPH (Figure 4, Tables S3–S5). The major sesquiterpene products of rice plants infested with BPH were β-ionone, β-ionone epoxide, E-β-farnesene, and linalool (Figure 5). E-β-farnesene (EBF) is the only major product that the BPH-resistant varieties RH and IL283 emitted in significantly higher amounts than the BPH-susceptible variety KD. Although RH emitted EBF at a lower level in the control condition, the emission was higher than KD in all the BPH treatment conditions. In addition, even though IL283 emitted EBF at a similar level to KD in the first six BPH feeding days, it started to emit at a higher level from day 7 to day 10 of BPH feeding. As RH and KD contain variations in *OsSTPS2* at both the genomic and protein product levels, and as the KD allele seems to lose its function because of its disrupted protein chain, it may be assumed that the reduced EBF production in KD was the result of this abnormality. Our results suggest that, besides several sesquiterpenes, EBF is the major product of this gene, and the gene was named “E-β-farnesene synthase (*OsEBFS*)” due to its prominent property of producing EBF as the volatile compound induced by BPH infestation.

The role of BPH-induced volatile compounds in the defense mechanisms of rice has long been studied [2,13,14]; however, only one sesquiterpene synthase gene (LOC_Os08g07100) has been reported to be induced by BPH [15]. Moreover, not only BPH feeding but also fall armyworm caterpillar infestation induces this gene; the major volatile products were found to be zingiberene and β-sesquiphellandrene, respectively [16]. In this present study, we demonstrated that the major volatile compound product of *OsSTPS2*, the novel BPH-feeding-induced sesquiterpene synthase, is EBF. We showed that the expression of the gene is controlled at the transcription level. Moreover, we also showed that two different processes can lead to the lesions in the protein product of the gene.

EBF is a common constituent of the aphid alarm phenomone [17,18,19] and has been reported to be used by wild potato as a repellent against aphid attack [20]. Recently, the role of EBF in the indirect defense mechanism has been studied in transgenic Arabidopsis [21]. Unfortunately, no evidence of its role in the defense mechanism of transgenic plants against aphids has been found. This may be due to the gene having been studied in different host plants with different insect enemies. Our finding that rice emitted EBF in response to BPH attack may open a path to understanding how rice communicates with its attackers.

## 3. Materials and Methods

### 3.1. Plant Materials and BPH Treatment

Based on their resistance to BPH, 20 rice varieties/lines were chosen for this study. Some rice varieties contain known BPH resistance genes. Mudgo (*BPH1*) and ASD7 (*BPH2*) [22], RH (*BPH3*) from Sri Lanka [23], PTB33 (*BPH3, BPH32*) [24], ARC10550 (*BPH5*), and Pokkali (*BPH9*) from India [25,26] were provided from the International Rice Research Institute. For KD-introgression-lines (IL) inherited *BPH3* gene from RH, UBN3078-101-342-4-143; IL143, UBN3078-101-342-4-162; IL162, UBN3078-101-342-4-283; IL283, UBN3078-101-342-6-302; IL302 and UBN3078-432-6-308; and IL308 [27] were selected. PL2 is the elite Thai BPH-resistant rice variety. The moderately susceptible elite Thai rice variety was PK3. The elite BPH-susceptible Thai rice varieties were PK2, HCS, RBR, JHN, and KD. Nipponbare and TN1 were the susceptible check from Japan and China, respectively.

Eighteen rice varieties/lines including ARC10550, ASD7, HCS, IL143, IL162, IL302, IL308, JHN, Mudgo, Nipponbare, PK2, PK3, PL2, Pokkali, PTB33, RBR, RH, and TN1 were used for the expression analysis of the *OsSTPS2* gene. Five rice varieties/lines, including ASD7, IL308, JHN, Mudgo, and Pokkali, were utilized for the antixenosis BPH resistance mechanism study. Finally, three rice varieties/lines, including IL283, KD, and RH, were used for plant volatile compound analysis.

The UBN-BPH population was used for BPH infestation in this study. The insects were grown on 7–10-day-old seedlings of TN1 in a temperature control room. Second- to third-instar nymphs were used for BPH treatment of the rice plants. The rice plants were sown by row in a seedbox, with 30 seeds each row, separated by 5 cm between rows. The seedboxes of 2-week-old seedlings were transferred to a cage for BPH feeding treatment 1 day before the test to allow the plant to settle into the experimental environment. After that, 5 to 10 nymphs per plant were released into the cage, and BPH feeding was conducted for 48 h. For control conditions, the same procedures were applied, except no BPHs were released into the cage for testing.

### 3.2. Genomic and Expression Analysis of OsSTPS2

Total RNA was isolated from the shoots and leaves of tested rice plants using the Trizol method (Invitrogen). The expression of *OsSTPS2* was analyzed via RT-PCR using the Titan One Tube RT-PCR System (Roche). The primers used were 5′-TGGACGACACATTTGATTCC-3’ (forward) and 5′-TAGGAACAGGATTGACGAAG-3′ (reverse). The PCR products were submitted to electrophoresis on 1% agarose gel at 100 V for 1 h before UV detection of the product bands. The rice ubiquitin (*OsUbq*) gene (forward primer 5’- ACCAGGACAAGATGATCTGCC-3′ and reverse primer 5′- AAGAAGCTGAAGCATCCAGC-3′) was used as the housekeeping gene check.

Genomic DNA was isolated from the rice varieties tested using DNA Trap (DNA Technology), and PCR fragments were amplified, sequenced, and aligned. The primers used for amplification and sequencing were 5′- CCCTCCACTGCACGGTCCCT-3′ (forward) and 5′-TGGTGCACGCGTGGGGGATA-3′ (reverse) for the 5′ upstream region and 5′-CCATGGAAGCAGTCAGGTTT-3′ (forward) and 5′ GACGGTAGGCATGGGTAATG-3′ (reverse) for exon 4 to exon 6 of *OsSTPS2*. The PCR products of both genomic and cDNA were isolated from agarose gel and sequenced (First BASE, Singapore). The sequences of all rice varieties were analyzed by alignment using ClustalW (https://www.ebi.ac.uk/Tools/msa/clustalo/; accessed on 21 May 2021).

### 3.3. BPH Resistance Evaluation

Five rice varieties/lines, JHN, Mudgo, ASD7, Pokkali, and IL308, were evaluated for BPH resistance under greenhouse conditions. Second- to third-instar nymph BPHs were released on 7-day-old rice seedlings at the rate of 8–10 insects per plant. Reactions of the seedlings to BPH were recorded as the damage score at 7 to 10 days after infestation (DAI) when the susceptible control Taichung Native1 (TN1) had completely died. The Standard Evaluation System for Rice (SES) [28] was applied to estimate the rice damage caused by BPH infestation.

### 3.4. Antixenosis BPH Resistance Mechanism

The antixenosis of the BPH feeding preference (AFP) was assessed by comparing seven combinations of five rice varieties/ lines: JHN, Mudgo, ASD7, Pokkali, and IL308 (Table 2). Seven-day-old seedlings of two different varieties/lines were placed in a 12 ounce translucent plastic cup. Ten third-instar nymphs were released into the plastic cup, and the numbers of nymphs that gathered on each seedling were counted at 1 h intervals until 8 h on the first day; subsequently, the numbers of nymphs were counted at 24, 48, 72, and 96 h.

### 3.5. Plant Volatile Collection and Identification

The rice varieties RH, KD, and IL283 were used to assess BPH-feeding-induced volatile emissions. Volatile compounds were extracted via solid-phase microextraction (SPME) from 4-week-old rice plants infested by BPH and kept under control conditions for 10 days.

Five grams of rice leaves was placed in a 250 mL Duran bottle. Extraction was performed at 75 °C with equilibrium and adsorption times of 20 and 30 min, respectively. The SPME sampling apparatus included an SPME fiber assembly holding a 1.0 cm fused-silica fiber (Supelco, Bellefonte, PA) with a 50/30 µm divinylbenzene/carboxen/polydimethysiloxane (DVB/CAR/PDMS) fiber used for sesquiterpene extraction. The fibers were thermally conditioned prior to adsorption at 230 °C in an injection port of the gas chromatography (GC) apparatus for 30 min to reduce bleeding and to prevent sample contamination.

Gas chromatography–mass spectrometry (GC-MS) analysis was performed using an HP model 6890 gas chromatograph (Agilent Technologies, Palo Alto, CA, USA) coupled to an HP model 5973 mass-selective detector. The capillary column was an AT-5MS (5% phenylmethylpolysiloxane) column (30 m × 0.25 mm i.d., 0.25 μm film thickness (Agilent Technologies, Palo Alto, CA, USA)). The oven temperature was initially held at 45 °C, and then increased at a rate of 2 °C/min to a final temperature of 230 °C, which was maintained for 13 min. The injector temperature was 230 °C. Purified helium was used as the carrier gas at a flow rate of 1 mL/min. EI mass spectra were collected at 70 eV ionization voltages over the range of 29–550 m/z. The electron multiplier voltage was 1150 V. The ion source and quadrupole temperatures were set at 230 °C and 150 °C, respectively. Identification of volatile components was performed by the comparison of their Kovát retention indices relative to C8 to C22 n-paraffin hydrocarbon mixtures and by the comparison of the mass spectra of individual components with the reference mass spectra in the Wiley 275 and NIST 98 database.

## Figures and Tables

**Table 1:** Haplotype pattern, *OsSTPS2* gene expression, and antixenosis mechanisms of rice varieties.

Rice Varieties	BPH Resistance Gene (s)	Haplotype Patterns	*OsSTPS2* Haplotype					Expression of *OsSTPS2*	7-Amino-Acid Insertion/Deletion	Antixenosis Mechanism
			ATHB-1	SBE1	P Element	SNP in EXON5	21-bp InDel			
KD	S	HP1	C	CA	T	CA	In	No	-	na
PK3	S		C	CA	T	CA	In	No	-	na
RBR	S		C	CA	T	CA	In	No	-	na
HCS	S		C	CA	T	CA	In	No	-	na
PK2	S		C	CA	T	CA	In	No	-	na
JHN	S	HP2	A	CG	C	TG	Del	Yes	Del	No AFP
Nipponbare	S		A	CG	C	TG	Del	Yes	Del	na
TN1	S		A	CG	C	TG	Del	Yes	Del	na
Mudgo	BPH1		A	CG	C	TG	Del	Yes	Del	No AFP
ASD7	BPH2		A	CG	C	TG	Del	Yes	Del	No AFP
ARC10550	BPH5		A	CG	C	TG	Del	Yes	Del	na
Pokkali	BPH9		A	CG	C	TG	Del	Yes	Del	No AFP
PTB33	BPH3, BPH32	HP3	A	TG	C	TG	In	Yes	In	na
RH	BPH3		A	TG	C	TG	In	Yes	In	na
IL143	BPH3		A	TG	C	TG	In	Yes	In	na
IL162	BPH3		A	TG	C	TG	In	Yes	In	na
IL302	BPH3		A	TG	C	TG	In	Yes	In	na
IL308	BPH3			TG	C	TG	In	Yes	In	AFP

S = susceptible, In = insertion, Del = deletion, na = not analyzed, AFP = antixenosis of BPH feeding preference.

**Table 2:** The list of antixenosis test treatments.

Treatment	Pairing	
	Left	Right
1	IL308	Mudgo
2	IL308	JHN
3	IL308	Pokkali
4	IL308	ASD7
5	JHN	Mudgo
6	JHN	Pokkali
7	JHN	ASD7

## Data Availability

Data is contained within the article or supplementary material.

## Supplementary Materials

The following are available online at https://www.mdpi.com/article/10.3390/plants10061049/s1, Figure S1: Association between four SNPs at 5’upstream and expression of *OsSTPS2* in 18 rice varieties. Figure S2: Sequence alignment analysis. Table S1: BPH damage score. Table S2: Antixenosis BPH feeding preference test. Table S3: Sesquiterpene products emitted by RH infested by BPH. Table S4: Sesquiterpene products emitted by IL283 infested by BPH. Table S5: Sesquiterpene products emitted by KD infested by BPH.
