# Supplementary material for: Rice Sesquiterpene Plays Important Roles in Antixenosis against Brown Planthopper in Rice
Source: Plants (Basel). 2021 May 22;10(6):1049. doi: 10.3390/plants10061049 (PMC8224800; doi:10.3390/plants10061049)
Supplement: Supplementary file 1 [file plants-10-01049-s001.zip › Supplementary Figure S1-S2.pdf]

Supplementary Figure

(A)

```
                                    ATHB-1
JHN                   TTTGACAAATTAATTATTGCTAGAACTGTTTCATGAATATTGTAATGTTATTTCACAAAG 358
ARC10550              TTTGACAAATTAATTATTGCTAGAACTGTTTCATGAATATTGTAATGTTATTTCACAAAG 353
ASD7                  TTTGACAAATTAATTATTGCTAGAACTGTTTCATGAATATTGTAATGTTATTTCACAAAG 353
TN1                   TTTGACAAATTAATTATTGCTAGAACTGTTTCATGAATATTGTAATGTTATTTCACAAAG 356
PTB33                 TTTGACAAATTAATTATTGCTAGAACTGTTTCATGAATATTGTAATGTTATTTCACAAAG 353
RH                    TTTGACAAATTAATTATTGCTAGAACTGTTTCATGAATATTGTAATGTTATTTCACAAAG 355
IL162                 TTTGACAAATTAATTATTGCTAGAACTGTTTCATGAATATTGTAATGTTATTTCACAAAG 356
IL302                 TTTGACAAATTAATTATTGCTAGAACTGTTTCATGAATATTGTAATGTTATTTCACAAAG 347
IL308                 TTTGACAAATTAATTATTGCTAGAACTGTTTCATGAATATTGTAATGTTATTTCACAAAG 357
Mudgo                 TTTGACAAATTAATTATTGCTAGAACTGTTTCATGAATATTGTAATGTTATTTCACAAAG 358
Pokkali               TTTGACAAATTAATTATTGCTAGAACTGTTTCATGAATATTGTAATGTTATTTCACAAAG 340
Nipponbare            TTTGACAAATTAATTATTGCTAGAACTGTTTCATGAATATTGTAATGTTATTTCACAAAG 343
IL143                 TTTGACAAATTAATTATTGCTAGAACTGTTTCATGAATATTGTAATGTTATTTCACAAAG 357
PK3                   TTTGACAAATTCATTATTGCTAGAACTGTTTCATGAATATTGTAATGTTATTTCACAAAG 356
RBR                   TTTGACAAATTCATTATTGCTAGAACTGTTTCATGAATATTGTAATGTTATTTCACAAAG 346
HCS                   TTTGACAAATTCATTATTGCTAGAACTGTTTCATGAATATTGTAATGTTATTTCACAAAG 358
PK2                   TTTGACAAATTCATTATTGCTAGAACTGTTTCATGAATATTGTAATGTTATTTCACAAAG 355
                      *********** ************************************************
```

(B)

```
                           SBF-1
JHN                   CTTTGCGTGGTTAATAATCTCTTTTTGATAACTATAATAAATCAAACTCGTGAATCCAAC 418
ARC10550              CTTTGCGTGGTTAATAATCTCTTTTTGATAACTATAATAAATCAAACTCGTGAATCCAAC 413
ASD7                  CTTTGCGTGGTTAATAATCTCTTTTTGATAACTATAATAAATCAAACTCGTGAATCCAAC 413
TN1                   CTTTGCGTGGTTAATAATCTCTTTTTGATAACTATAATAAATCAAACTCGTGAATCCAAC 416
PTB33                 CTTTGTGTGGTTAATAATCTCTTTTTGATAACTATAATAAATCAAACTCGTGAATCCAAC 413
RH                    CTTTGTGTGGTTAATAATCTCTTTTTGATAACTATAATAAATCAAACTCGTGAATCCAAC 415
IL162                 CTTTGTGTGGTTAATAATCTCTTTTTGATAACTATAATAAATCAAACTCGTGAATCCAAC 416
IL302                 CTTTGTGTGGTTAATAATCTCTTTTTGATAACTATAATAAATCAAACTCGTGAATCCAAC 407
IL308                 CTTTGTGTGGTTAATAATCTCTTTTTGATAACTATAATAAATCAAACTCGTGAATCCAAC 417
Mudgo                 CTTTGCGTGGTTAATAATCTCTTTTTGATAACTATAATAAATCAAACTCGTGAATCCAAC 418
Pokkali               CTTTGCGTGGTTAATAATCTCTTTTTGATAACTATAATAAATCAAACTCGTGAATCCAAC 400
Nipponbare            CTT-GCGTGGTTAATAATCTCTTTTTGATAACTATAATAAATCAAACTCGTGAATCCAAC 402
IL143                 CTTTGTGTGGTTAATAATCTCTTTTTGATAACTATAATAAATCAAACTCGTGAATCCAAC 417
PK3                   CTTTGCATGGTTAATAATCTCTTTTTGATAACTATAATAAATCAAACTCGTGAATCCAAC 416
RBR                   CTTTGCATGGTTAATAATCTCTTTTTGATAACTATAATAAATCAAACTCGTGAATCCAAC 406
HCS                   CTTTGCATGGTTAATAATCTCTTTTTGATAACTATAATAAATCAAACTCGTGAATCCAAC 418
PK2                   CTTTGCATGGTTAATAATCTCTTTTTGATAACTATAATAAATCAAACTCGTGAATCCAAC 415
                      *** *  *****************************************************
```

(C)

```
                                      P
JHN                          CAACCAACCAGAGATCGGAAGAATGGATGAGGAAGAGAGCTGACGAGCTCAAGGAGAAAGTCCG 478
ARC10550                     CAACCAACCAGAGATCGGAAGAATGGATGAGGAAGAGAGCTGACGAGCTCAAGGAGAAAGTCCG 473
ASD7                         CAACCAACCAGAGATCGGAAGAATGGATGAGGAAGAGAGCTGACGAGCTCAAGGAGAAAGTCCG 473
TN1                          CAACCAACCAGAGATCGGAAGAATGGATGAGGAAGAGAGCTGACGAGCTCAAGGAGAAAGTCCG 476
PTB33                        CAACCAACCAGAGATCGGAAGAATGGATGAGGAAGAGAGCTGACGAGCTCAAGGAGAAAGTCCG 473
RH                           CAACCAACCAGAGATCGGAAGAATGGATGAGGAAGAGAGCTGACGAGCTCAAGGAGAAAGTCCG 475
IL162                        CAACCAACCAGAGATCGGAAGAATGGATGAGGAAGAGAGCTGACGAGCTCAAGGAGAAAGTCCG 476
IL302                        CAACCAACCAGAGATCGGAAGAATGGATGAGGAAGAGAGCTGACGAGCTCAAGGAGAAAGTCCG 467
IL308                        CAACCAACCAGAGATCGGAAGAATGGATGAGGAAGAGAGCTGACGAGCTCAAGGAGAAAGTCCG 477
Mudgo                        CAACCAACCAGAGATCGGAAGAATGGATGAGGAAGAGAGCTGACGAGCTCAAGGAGAAAGTCCG 478
Pokkali                      CAACCAACCAGAGATCGGAAGAATGGATGAGGAAGAGAGCTGACGAGCTCAAGGAGAAAGTCCG 460
Nipponbare                   CAACCAACCAGAGATCGGAAGAATGGATGAGGAAGAGAGCTGACGAGCTCAAGGAGAAAGTCCG 462
IL143                        CAACCAACCAGAGATCGGAAGAATGGATGAGGAAGAGAGCTGACGAGCTCAAGGAGAAAGTCCG 477
PK3                          CAACTAACCAGAGATCGGAAGAATGGATGAGGAAGAGAGCTGACGAGCTCAAGGAGAAAGTCCG 476
RBR                          CAACTAACCAGAGATCGGAAGAATGGATGAGGAAGAGAGCTGACGAGCTCAAGGAGAAAGTCCG 466
HCS                          CAACTAACCAGAGATCGGAAGAATGGATGAGGAAGAGAGCTGACGAGCTCAAGGAGAAAGTCCG 478
PK2                          CAACTAACCAGAGATCGGAAGAATGGATGAGGAAGAGAGCTGACGAGCTCAAGGAGAAAGTCCG 475
                             **** ***********************************************************
```

Supplementary Figure S1. Association between 4SNP at 5'upstream and expression of OsSTPS2 in 18 rice cultivars. Sequence alignment at ATHB-1 (A), SBF-1 (B) and P (C) transcription factor binding sites, consensus sequence of these elements are labeled in red color. The SNPs found in each element are labeled in blue color.

(A)

```
KD                        AAACTGTCCACCTACTATCTCCAGGAAGCCAAATGGTCACACCAGAGGCATAAACCAAGC
PK3                       AAACTGTCCACCTACTATCTCCAGGAAGCCAAATGGTCACACCAGAGGCATAAACCAAGC
HCS                       AAACTGTCCACCTACTATCTCCAGGAAGCCAAATGGTCACACCAGAGGCATAAACCAAGC
PK2                       AAACTGTCCACCTACTATCTCCAGGAAGCCAAATGGTCACACCAGAGGCATAAACCAAGC
RBR                       AAACTGTCCACCTACTATCTCCAGGAAGCCAAATGGTCACACCAGAGGCATAAACCAAGC
Mudgo                     AAACTGTCCACCTACTATCTCCAGGAAGCTGAA---------------------CCAAGC
TN1                       AAACTGTCCACCTACTATCTCCAGGAAGCTGAA---------------------CCAAGC
Pokkali                   AAACTGTCCACCTACTATCTCCAGGAAGCTGAA---------------------CCAAGC
ARC10550                  AAACTGTCCACCTACTATCTCCAGGAAGCTGAA---------------------CCAAGC
ASD7                      AAACTGTCCACCTACTATCTCCAGGAAGCTGAA---------------------CCAAGC
JHN                       AAACTGTCCACCTACTATCTCCAGGAAGCTGAA---------------------CCAAGC
PTB33                     AAACTGTCCACCTACTATCTCCAGGAAGCTGAATGGTCACACCAGAGGCATAAACCAAGC
IL308                     AAACTGTCCACCTACTATCTCCAGGAAGCTGAATGGTCACACCAGAGGCATAAACCAAGC
IL143                     AAACTGTCCACCTACTATCTCCAGGAAGCTGAATGGTCACACCAGAGGCATAAACCAAGC
IL162                     AAACTGTCCACCTACTATCTCCAGGAAGCTGAATGGTCACACCAGAGGCATAAACCAAGC
IL302                     AAACTGTCCACCTACTATCTCCAGGAAGCTGAATGGTCACACCAGAGGCATAAACCAAGC
Nipponbare                AAACTGTCCACCTACTATCTCCAGGAAGCTGAA---------------------CCAAGC
                          *****************************  **                     ******
```

(B)

```
KD                        AAGCTGAA---------------------CCAAGCTTCGGTGACCAGATTACTTT
JHN                       AAGCTGAA---------------------CCAAGCTTCGGTGACCAGATTACTTT
TN1                       AAGCTGAA---------------------CCAAGCTTCGGTGACCAGATTACTTT
Mudgo                     AAGCTGAA---------------------CCAAGCTTCGGTGACCAGATTACTTT
Pokkali                   AAGCTGAA---------------------CCAAGCTTCGGTGACCAGATTACTTT
ARC10550                  AAGCTGAA---------------------CCAAGCTTCGGTGACCAGATTACTTT
ASD7                      AAGCTGAA---------------------CCAAGCTTCGGTGACCAGATTACTTT
Nipponbare                AAGCTGAA---------------------CCAAGCTTCGGTGACCAGATTACTTT
IL143                     AAGCTGAATGGTCACACCAGAGGCATAAACCAAGCTTCGGTGACCAGATTACTTT
IL302                     AAGCTGAATGGTCACACCAGAGGCATAAACCAAGCTTCGGTGACCAGATTACTTT
IL162                     AAGCTGAATGGTCACACCAGAGGCATAAACCAAGCTTCGGTGACCAGATTACTTT
PTB33                     AAGCTGAATGGTCACACCAGAGGCATAAACCAAGCTTCGGTGACCAGATTACTTT
PL2                       AAGCTGAATGGTCACACCAGAGGCATAAACCAAGCTTCGGTGACCAGATTACTTT
IL308                     AAGCTGAATGGTCACACCAGAGGCATAAACCAAGCTTCGGTGACCAGATTACTTT
RH                        AAGCTGAATGGTCACACCAGAGGCATAAACCAAGCTTCGGTGACCAGATTACTTT
                          ********                     **************************
```

(C)

```
JHN               EFQKLSTYYLQEAE-------PSFGDQITLTAMSSVIPL
TN1               EFQKLSTYYLQEAE-------PSFGDQITLTAMSSVIPL
Mudgo             EFQKLSTYYLQEAE-------PSFGDQITLTAMSSVIPL
Pokkali           EFQKLSTYYLQEAE-------PSFGDQITLTAMSSVIPL
ARC10550          EFQKLSTYYLQEAE-------PSFGDQITLTAMSSVIPL
ASD7              EFQKLSTYYLQEAE-------PSFGDQITLTAMSSVIPL
Nipponbare        EFQKLSTYYLQEAE-------PSFGDQITLTAMSSVIPL
IL143             EFQKLSTYYLQEAEWSHQRHKPSFGDQITLTAMSSVIPL
IL302             EFQKLSTYYLQEAEWSHQRHKPSFGDQITLTAMSSVIPL
IL162             EFQKLSTYYLQEAEWSHQRHKPSFGDQITLTAMSSVIPL
PTB33             EFQKLSTYYLQEAEWSHQRHKPSFGDQITLTAMSSVIPL
PL2               EFQKLSTYYLQEAEWSHQRHKPSFGDQITLTAMSSVIPL
IL308             EFQKLSTYYLQEAEWSHQRHKPSFGDQITLTAMSSVIPL
RH                EFQKLSTYYLQEAEWSHQRHKPSFGDQITLTAMSSVIPL
                  **************       ******************
```

Supplementary Figure S2. Sequence alignment analysis. genomic (A), cDNA (B), amino acid (C) sequence alignments of 2SNPs, 21-bp, and 7-amino acid deletion respectively in several rice cultivars. A; The TG/CA SNPs in exon 5 genomic DNA is labeled by blue/red color. B and C; the rice cultivars with 21-bp and 7-amino-acid deletion are labeled in red color.
